# Measurement of betamethasone concentration in maternal serum treated for fetal lung maturity; Is it feasible?

**DOI:** 10.1186/s12958-016-0142-4

**Published:** 2016-02-10

**Authors:** Raed Salim, Abeer Suleiman, Raul Colodner, Zohar Nachum, Lee H. Goldstein, Eliezer Shalev

**Affiliations:** Department of Obstetrics and Gynecology, Emek Medical Center, Afula, 18101 Israel; Rappaport Faculty of Medicine, Technion, Haifa, Israel; Clinical Microbiology Laboratory, Emek Medical Center, Afula, Israel; Department of Internal Medicine C, Clinical Pharmacology and toxicology Unit, Emek Medical Center, Afula, Israel

**Keywords:** Betamethasone concentration, ELISA kit, Preterm birth, Respiratory distress syndrome

## Abstract

**Background:**

The association between maternal serum concentration of betamethasone given for fetal lung maturity and perinatal outcome has not been investigated. This may be due to an absence of a reliable method for measuring serum betamethasone concentrations. We aimed in the current study to assess the feasibility of a specific ELISA kit to measure the concentrations of betamethasone in maternal serum and to examine the trend of sequential measurements after a course of betamethasone for fetal lung maturity.

**Methods:**

Pregnant women at risk for preterm birth who received betamethasone between 24 and 34 weeks of gestation were prospectively included. Serum concentrations were determined before administering betamethasone (baseline), and 36 hours, 48 hours, 72 hours, and 5 to 7 days after the 1^st^ dose. Betamethasone concentration in samples was determined using Corticosteroid ELISA kit. The Friedman test was used to test whether there were significant differences between the measurements.

**Results:**

Five singleton pregnancies were included. Using the ELISA kit, betamethasone concentration in maternal serum samples was obtained for all women. Among the five measurements performed, the concentration was highest at 36 hours after the 1^st^ dose and close to baseline at the 5^th^ measurement performed after 5 to 7 days (*p* < 0.05). Serum concentration varied at each time point between the five women but similar trend was observed.

**Conclusion:**

Betamethasone concentration is measurable in the serum of pregnant women with this ELISA kit.

## Background

Preterm birth is a major contributor to perinatal mortality and morbidity and affects approximately 7 to 12% of births in developed countries [1]. It is responsible for approximately 75% of all neonatal deaths and 50% of childhood neurological morbidities. Additionally, these premature infants are at high risk for acute medical complications including respiratory distress syndrome, intraventricular hemorrhage, and necrotizing enterocolitis [2, 3].

Liggins’ 1969 study with sheep that found improved survival for preterm lambs delivered after corticosteroid exposure [4], paved the way for one of the most important developments in antenatal care for women at risk for preterm birth [5]. Subsequent randomized controlled trials, which demonstrated reduced perinatal mortality and morbidity, particularly respiratory distress syndrome, led to a change in antenatal care, and antenatal betamethasone therapy has become a standard of care in the majority of medical centers worldwide [6, 7].

The currently accepted dosage of betamethasone was determined by Liggins and Howie in the 1970s; since that time, pregnant women at risk for preterm birth have been administered a fixed dosage of betamethasone, regardless of gestational age, maternal body mass index, and the presence of a singleton or multiple gestation [8]. Although betamethasone treatment reduces mortality and morbidity in general [9], its effect for a particular woman may be unpredictable, and varying outcomes between two preterm neonates with similar obstetrical background born at a similar gestational age after treatment with betamethasone may often be observed. Moreover, among multiple pregnancies, the benefit of betamethasone is uncertain [10, 11]. For that reason the optimal dosage for betamethasone in terms of benefit and safety is still unknown.

To the best of our knowledge, the relationship between betamethasone concentration and perinatal outcome has not been reported. In part, this may be due to an absence of a reliable method for measuring serum betamethasone concentrations. In view of that, we aimed in this study to assess the ability of a specific ELISA kit to measure the concentrations of betamethasone in maternal serum and to examine the trend of sequential measurements in maternal serum after a complete course of betamethasone for lung maturity.

## Methods

This prospective study was held at a university teaching medical center between July 2012 and April 2014. Pregnant women between 24 weeks 0 days and 33 weeks 6 days of gestation, had singleton gestation, and received a complete course of betamethasone due to threatened preterm birth were included. A complete course of betamethasone (consisting of 50% betamethasone phosphate and 50% betamethasone acetate) involved two 12 mg intramuscular doses 24 hours apart. Women who received an incomplete course of betamethasone because of failure to delay delivery or who received corticosteroids for other reasons during pregnancy, had multiple gestations, or fetal malformations diagnosed in the antepartum period were excluded from the study.

In order to examine whether betamethasone levels are measurable and in order to examine the dynamics of betamethasone concentrations against time from treatment, blood samples were drawn from each participating woman at five time points: immediately before administering the betamethasone (baseline); at 36 hours after the first dose; at 48 hours after the first dose; at 72 hours after the first dose, and 5 to 7 days after the first dose, given that a preterm birth did not occur earlier. These consecutive measurements were chosen in order to examine whether the trend of concentrations acted comparably to the anticipated clinical effect of betamethasone, which is best achieved within 7 days from the initial dose [9].

In order to examine whether half-life (t1/2) of betamethasone concentration varies during different periods after betamethasone administration and between the participating women, t1/2 of betamethasone was calculated between the second and third measurements (36 to 48 hours after the first injection) and between the third and fourth measurements (48 to 72 hours after the first injection) for all women. Calculation was performed using the formula t1/2 = t * ln(2)/ln(N0/Nt) where, t = time between measurements, ln = natural logarithm, N0 = concentration at the beginning, and Nt = concentration at the end of the time between the two measurements.

### Determining betamethasone concentrations

Serum samples were kept at -70°C until processing. Betamethasone concentration in samples was determined using the Corticosteroid ELISA kit (Randox Laboratories Ltd., Crumlin, BT29, UK) according to the manufacturer’s instructions, processed in a Triturus apparatus (Grifols Diagnostics, Parets del Valles, Spain). This kit, originally designed for urine, tissue, and serum samples in the veterinary field, was evaluated in our laboratory for use with human samples. Evaluation included precision, repeatability, linearity, and analytical sensitivity (lower limit of detection). Those parameters were analyzed by spiking betamethasone (provided with the kit) and the same generic betamethasone that was administered to women into a pool of human sera. According to linearity range obtained in the evaluation, study samples were diluted accordingly in order to be measured within this range.

The present study was approved by the ethics committee of Emek Medical Center in compliance with the Helsinki declaration. Signed consent was obtained from each of the participating women before receiving the first betamethasone injection.

### Statistical analysis

The mean and standard deviation of the betamethasone levels from all samples were calculated. The Friedman test was used to determine whether there were significant differences between betamethasone concentrations. Significance level was set at *p* < 0.05. For continuous variables, the Student *t*-test was implemented. SAS 9.2 was used to perform the statistical analyses and the graphical presentation (SAS Institute Inc., Cary, NC, USA).

## Results

A total of eight women were approached and consented. Three women delivered before receiving a complete course of betamethasone and for that reason they were excluded. Overall, five women (A to E) received a complete course of betamethasone and were included in the study. Demographic and obstetric parameters are presented in Table 1. Base line and last measurements of woman E were not performed; the first was due to technical issues and the last due to the fact that she delivered before the scheduled examination. Among all women, two delivered preterm, woman B and woman E (Table 1). Woman B delivered at 30 weeks of gestation, 3 weeks after betamethasone administration. Betamethasone concentration 48 hours after the first dose was 0.64 ng/mL. The neonate developed respiratory distress syndrome. Woman E delivered at 29 weeks of gestation, less than a week after betamethasone administration. Betamethasone concentration 48 hours after the first dose was 1.61 ng/mL. The neonate did not develop respiratory distress syndrome. The other 3 women delivered at term. None of the neonates developed intraventricular hemorrhage or necrotizing enterocolitis.

**Table 1:** Demographic and obstetrical parameters of the participating women

Woman	Age (years)	Body mass index (kg/m^2^)^a^	Parity	Gestational age at betamethasone treatment (weeks)	Indication for betamethasone treatment	Gestational age at delivery (weeks)	Respiratory distress syndrome
A	25	29.8	1	27.6	Preterm labor	37.1	No
B	25	27.5	0	27	Preterm labor	30.0	Yes
C	27	33.1	1	32.2	Preeclampsia	37.4	No
D	36	29.7	2	32.1	Preeclampsia	39.3	No
E	33	26.4	0	28.2	Preterm labor	29.0	No

^a^Calculated at the time of betamethasone treatment

**Fig. 1:**
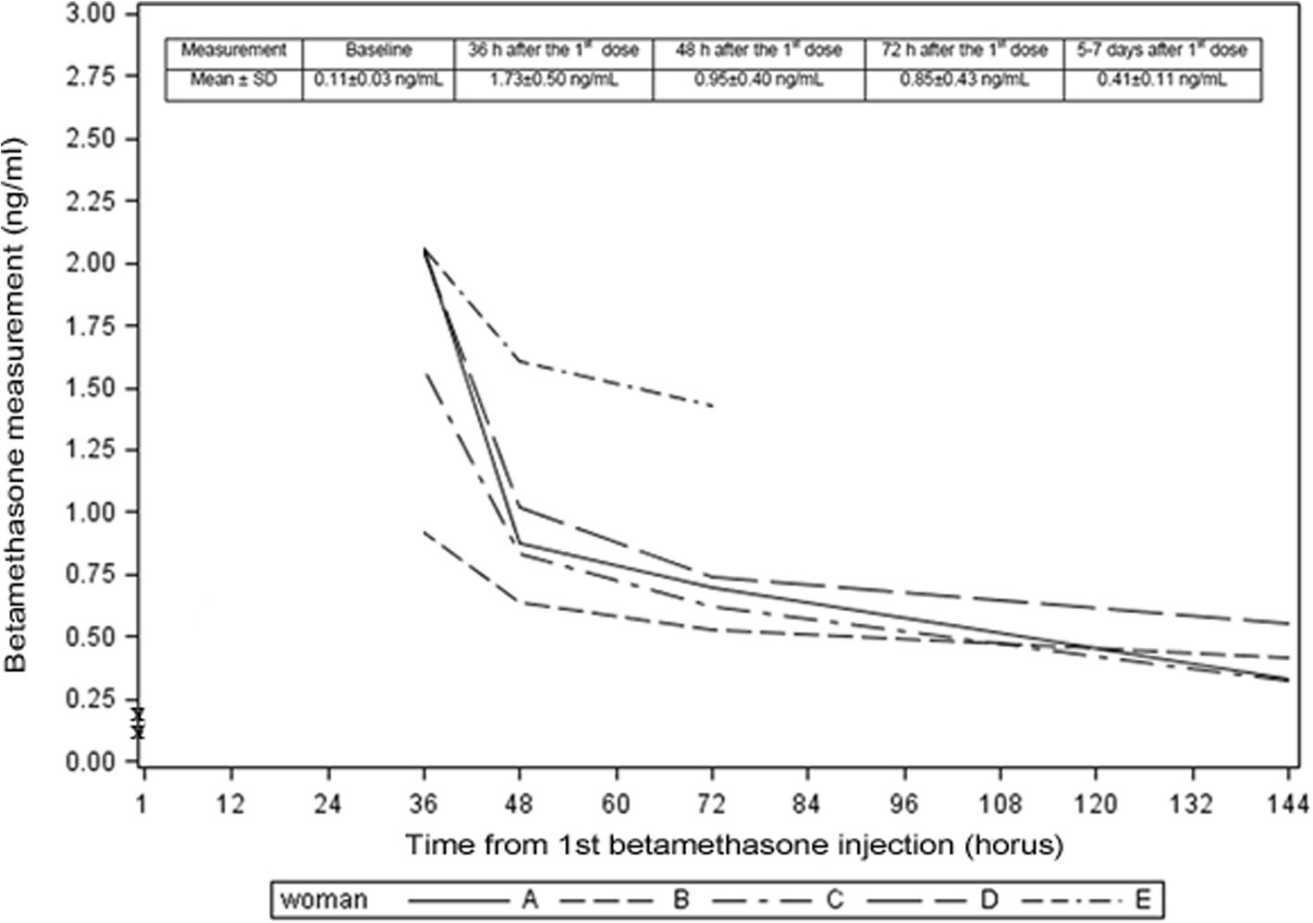
Trends of the sequential betamethasone concentration measurements of the 5 women included in the study

As shown in Fig. 1, betamethasone concentrations could be measured in the human serum samples. Among the measurements performed for each woman, the concentration was highest 36 hours after the first dose of betamethasone. The concentration diminished gradually to a level close to the baseline at the 5^th^ measurement performed 5 to 7 days after the first dose of betamethasone. The trend between measurements was significant (*p* < 0.05). Although the trend of sequential measurements was similar for the five women, there were obvious variations at each time point (Fig. 1).

Calculated half-life of betamethasone differed according to the time elapsed from its administration (Table 2). Mean half-life after 36 to 48 hours from administration (21.2 ± 10.6 hours) was significantly shorter compared to 48 to 72 hours after administration (82 ± 35.6 hours), (*p* = 0.006).

**Table 2:** Calculated half-life of betamethasone concentrations among the participating women

	Woman A	Woman B	Woman C	Woman D	Woman E	Mean±SD
T_1/2_, 36–48 hours after 1^st^ dose (hours)	9.0	22.9	28.5	12.0	33.7	21.2 ± 10.6
T_1/2_, 48–72 hours after 1^st^ dose (hours)	72.7	88.2	57.0	51.8	140.3	82 ± 35.6

Data are mean ± standard deviation. T_1/2,_ Half-life

The evaluation of betamethasone measurement kit performance demonstrated a high precision for spiked betamethasone in the human sera pool: average coefficient of variation of 3.5 and 6.1% for kit and generic betamethasone, respectively, for expected values between 0.1 and 50 ng/mL. The kit demonstrated a good linearity (average bias = ±4.0%) for the range between 0.1 and 50 ng/mL. Analytical sensitivity (lower limit of detection) was 0.05 ng/mL.

## Discussion

In this study we were able to measure betamethasone concentrations in the serum of pregnant women with the use of corticosteroid ELISA kit. Among the measurements performed for each woman, the concentration was highest at 36 hours after the first dose of betamethasone followed by a gradual decrease to a level close to the baseline at the 5^th^ measurement at 5 to 7 days. The trend of sequential measurements performed similarly between all women; however the concentration varied at each time point between the five women. The near to baseline concentration at 5 to 7 days is similar to the described perinatal clinical effect that is best achieved within 7 days after drug administration [9].

The National Institutes of Health and the American College of Obstetricians and Gynecologists recommend routine glucocorticoid administration to women at risk for preterm birth between 24 and 34 weeks gestation to accelerate fetal lung maturation and to decrease the incidence of other neonatal morbidity and mortality related to prematurity [7, 12]. When betamethasone is administered, the recommended regimen is two doses, 12 mg each, given intramuscularly 24 hours apart [7, 12].

Although betamethasone treatment reduces mortality and morbidity in general, its effect on a particular woman is usually unpredictable. The current dosage of betamethasone was determined by Liggins and Howie during the 1970s, and since then a fixed dosage is administered to all pregnant women regardless of gestational age, maternal body mass index, and whether a singleton or multiple gestation is present [8]. Additionally, betamethasone rapidly crosses the placenta [13]. Its administration to the pregnant mother inevitably exposes the fetus to glucocorticoid concentrations that may be inappropriate for the current stage of fetal development [14]. High blood pressure [15], reduced head circumference at birth [16], and reduced brain weight in monkey fetuses [17, 18] are among the several adverse effects reported after glucocorticoid given to enhance fetal lung maturity. In view of that, the optimum betamethasone dosage and concentration in terms of benefit and safety to the developing fetus at any gestational age has not been established.

Loehle et al. reported that routine therapy of two 12 mg doses of betamethasone phosphate alone given intramuscularly to the average weight mother 24 h apart is supramaximal for fetal lung maturation. Their data showed that half the normal clinical dose, adjusted for weight, may be functionally adequate [14]. On the other hand, Ballabh et al. reported that the efficacy of betamethasone to promote fetal lung maturation in twin pregnancy may be reduced since the dose of betamethasone is suboptimal [19]. They reported that the reasons are due to shorter half-life and greater clearance of betamethasone in twin pregnancy than in singleton pregnancy. This may be attributed to the presence of two fetoplacental units in twins, which causes greater metabolism of betamethasone than in singletons [19], or a dilution effect due to the increased maternal blood volume and total body water [20]. In contrast to the previous studies we show that there is a variation in the concentrations among women with singleton pregnancies and a fixed dosage, whether smaller or larger than the acceptable regimen, may not be sufficient to reduce perinatal complications among all women.

The half-lives of betamethasone concentration found in the current study differ from the fixed 9.0 ± 2.7 hours reported by Ballabh et al. [19]. This may be due to imprecision in the technique used to measure betamethasone concentrations. Ballabh et al. used a radioimmunoassay that was originally developed to measure dexamethasone and was modified to measure betamethasone concentration [19]. Additionally, and compared to their results, two different half-lives were noted in the current study. Sahin et al. reported that for most drugs there is no single half-life that can be readily accepted. This is due to the fact that the systemic concentration time profile for most drugs is best described by a multi-exponential function, thereby yielding more than one half-life to describe the drug. The choice of a single appropriate half-life value for such drugs is unclear [21]. This may also be explained with the two-compartment model that adds a peripheral compartment to the one-compartment model. The peripheral compartment represents the opportunity for a drug to leave the plasma and enter other tissues. Among pregnant women, a third compartment may be present – the fetoplacental. Drugs transfer to the fetus across the placenta and elimination from the fetus is by diffusion back to the maternal compartment. Moreover, the fetus plays an important role in the equilibrium of a compound because the fetus swallows amniotic fluid and urinates; there is a sort of drug recirculation within the fetal compartment along with the transfer back to maternal blood [22]. This may explain the existence of more than one half-life found in the current study.

## Conclusions

The results of the current study do not provide evidence as to the precise dosage of betamethasone that needs to be administered; the minimum betamethasone dosage that would be clinically effective is yet to be determined. Basic pharmacologic principles suggest that the drug concentration in maternal serum may determine the fetal response and perinatal outcome and, as such, the optimal treatment regimen. Given that, the results of the current study may pave the way for future studies that could determine the optimum concentration and, accordingly, the dosage of betamethasone by investigating the minimum concentration that would be clinically effective to positively affect perinatal outcome.
